# Ameliorating potential of *Auricularia auricula-judae* polysaccharides in mitigating hypercaloric diet-induced behavioral disorders through gut microbiota regulation

**DOI:** 10.3389/fnut.2025.1585778

**Published:** 2025-05-29

**Authors:** Yujie Fei, Jingwen Zhang, Sijia Yuan, Ye Liu, Xiaoru Wang, Meng Wang, Haoan Zhao, Qian Liu

**Affiliations:** ^1^College of Food Science and Technology, Northwest University, Xi'an, China; ^2^Xi'an Xida Institute of Life Sciences and Health Research Co., Ltd., Xi'an, China; ^3^Shaanxi Functional Food Engineering Center Co., Ltd., Xi'an, China

**Keywords:** *Auricularia auricula-judae*, polysaccharides, behavioral disorders, hypercaloric diet, gut microbiota

## Abstract

As a major nutraceutical component of a traditional edible fungus *Auricularia auricula-judae, Auricularia auricula-judae* polysaccharide (AAP) has been well-documented due to its outstanding hypolipidemic and hypoglycemic bioactivities. This study investigated the effects of AAP on hypercaloric diet-induced cognitive dysfunction in mice and the underlying mechanisms. Behavioral and histological results demonstrated that AAP could ameliorate high-fat and high-fructose diets (HFFD)-induced memory impairment and neuronal loss. AAP significantly inhibited inflammatory responses and balanced oxidative stress states in mice brain and colon tissues. AAP dietary supplements remarkably reshaped gut bacteria composition. The abundance of *Dubosiella, Bacteroides*, and *Parasutterella* were significantly increased. Differential bacteria abundance showed a strong correlation with behavioral related indicators, inflammatory factors, antioxidant enzymes and serum metabolites levels. These results suggest that AAP is able to ameliorate high-calorie diet-induced cognitive dysfunction in mice, as well as modulate the regulation of gut flora homeostasis and serum metabolites in mice. And these results are of positive significance for promoting the utilization of *Auricularia auricula-judae* resources and for the development of nutraceutical products in the brain with AAP as the primary active component.

## Graphical Abstract

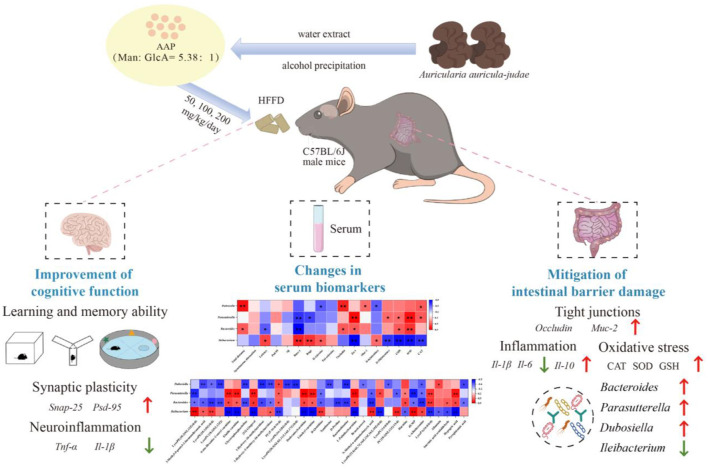

## 1 Introduction

Numerous researches have identified a substantial correlation between the intake of foods rich in saturated fats (high-fat diet, HFD) and development of cognitive impairment (1, 2). Both short- (1 week) and long-term (10 months) HFD interventions induce learning and memory decline in mice (3–5). The HFD modulates processes including oxidative stress, neuroinflammation, insulin resistance, synaptic plasticity dysfunction, blood-cerebrospinal fluid barrier dysfunction, and impaired cerebral blood flow that have been identified as key pathophysiological mechanisms leading to cognitive impairment (6, 7). There are strong correlations between intestinal flora and cognition. Altering the composition of the host gut microorganisms by using antibiotic or fecal transplants can modulate host behaviors such as learning, memory, mood, anxiety, depression, and stress (8, 9). Promoting homeostasis of the gut flora-gut-brain axis has emerged an effective measure to improve brain nutritional status and cognitive function.

*Auricularia Auricula-judae*, which is rich in gelatin, is a common and delicious health food that is widely available, is referred to as “black treasure”, and is known to be an “intestinal scavenger”(10). *Auricularia Auricula-judae* polysaccharide (AAP) is a crucial nutraceutical component in the *Auricularia Auricula-judae* fruiting body, for it can promote intestinal peristalsis, lower blood glucose and blood lipids, and exert antioxidant, anti-thrombosis, and immunity-enhancing effects (11, 12). The main components of AAP are glucose, mannose, and galactose under hot water extraction conditions (13, 14); the structural backbone of AAP involves a (1 → 4)-D-glucopyranosyl group and an O6 glucopyranose side group, with a glucuronic acid structure extracted by 70% ethanol solution (15). Several researches suggest that AAP may have a beneficial effect on brain health. Xiong et al. found that AAP can increase the number of hippocampal neurons surviving in brain tissue, and significantly increase superoxide dismutase (SOD) activity, which can protect against ischemic brain damage in neonatal rats to a certain degree (16). The wood ear mushroom-containing polyherbal GCJ attenuated cerebral ischemia-reperfusion injury-induced cognitive deficits in MetS rats, potentially linked to its modulation of AChE activity and activation of eNOS, BDNF, and pERK/ERK pathways in memory-related brain regions (17). Oral administration of *Auricularia polytricha* aqueous extract (AEAP, 400/600 mg/kg) significantly attenuated haloperidol-induced catalepsy in rats, which may be attributed to the modulation of oxidative stress by AEAP, such as the levels of superoxide dismutase, catalase (CAT), and glutathione (GSH) (18). Two hundreds mg/kg/day of purified fraction AAP I-a obtained by ultrasound-assisted extraction method administered by gavage for 35 days significantly reduced malondialdehyde (MDA, a lipid peroxidation product) levels in mouse brains, increased SOD activity and GSH content, and significantly inhibited D-galactose-induced aging in mice (19).

While accumulating evidence has substantiated the therapeutic potential of AAP in nutritional management, critical knowledge gaps persist regarding its mechanistic interplay with cognitive dysfunctions induced by obesogenic diets, particularly in elucidating how specific bioactive constituents counteract neural metabolic disturbances. This study aimed to examine the interventional impacts of AAP on cognitive dysfunction caused by high-calorie diets in mice and analyze the mechanism based on the intestinal flora-gut-brain axis using metabolomics and intestinal microbiomics techniques. The results will lay a theoretical foundation for further studies of intervention in high-calorie diet-induced nutritional health disorders using natural functional food polysaccharide components, and provide new ideas to guide individuals and societies in establishing reasonable dietary structures and addressing the issue of overnutrition.

## 2 Materials and methods

### 2.1 Preparation of AAP

A 30 g weighed sample of powdered *Auricularia Auricula-judae* (purchased from Shaanxi Tianmei Green Industry Co., Ltd.) was extracted by adding distilled water at 100°C to a material-liquid ratio of 1:50 (g:mL) for 2 h. The extraction solution was centrifuged at 2,800 × g for 15 min and the supernatant was collected and concentrated. Then, anhydrouas ethanol was added at a ratio of 1:4 (v/v), for ethanol precipitation. After 12 h of ethanol precipitation, the sample was centrifuged at 2,800 × g for 15 min for 10 min, and the filtered residue was retained and dissolved in water. Proteins were removed via the Sevag method by adding a reagent (dichloromethane:n-butanol = 4:1, v/v) to the solution, stirring for 20 min, and centrifuging at 2,800 × g for 15 min. The supernatant was removed, the precipitant and organic layers were discarded, and the procedure was repeated 4–5 times. AAP was obtained by dialysis in flowing tap water for 3 d using cellulose dialysis bags with a molecular cut-off of 3,000 Da, followed by concentration, freeze-drying, weighing, and bagging. The carbohydrate content, protein content, and uronic acid content of AAP were 51.24 ± 4.52%, 4.18 ± 0.36%, and 12.31 ± 3.26%, respectively. Mannose and glucuronic acid are the main monosaccharide existed in AAP, and the relative molar ration of the two is 5.38:1 (Supplementary Figure S1). Calculated according to the calibration curve, the average molecular weight of AAP was 1.91 × 10^3^ kDa (Supplementary Figure S2).

### 2.2 Animal treatments

**Figure 1:**
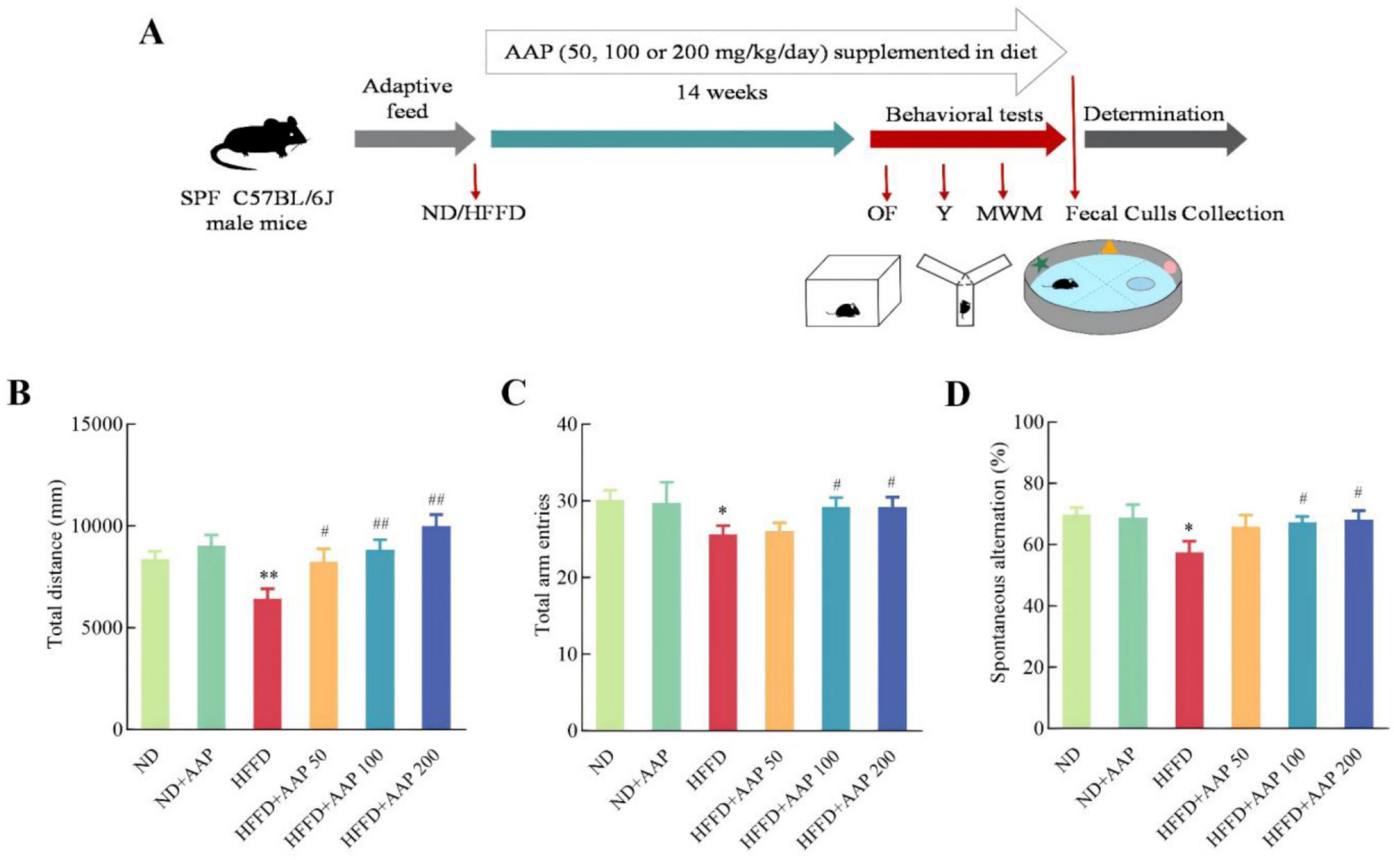
Timeline illustrating AAP treatment and evaluations of mouse cognitive functions. **(A)** Experimental scheme for effects of AAP on mouse memory loss induced by HFFD; **(B)** Total movement distance in open-field trials; **(C)** Total number of arm entries and **(D)** percentage of alternations in the Y maze. The data are presented as means ± SEM, *n* = 10. *p < 0.05, **p < 0.01, vs. ND group, ^#^*p* < 0.05, ^##^*p* < 0.01 vs. HFFD group.

Animal experiment protocols were approved by the Animal Ethics Committee of the Laboratory Animal Center of Northwest University (NWU-AWC-20200401M). Following a week of acclimation, 60 C57BL/6J mouse (SPF, 7-week-old, male, purchased from Xi'an Jiaotong University) were split into six groups randomly. A cognitive impairment model was induced by chronic exposure to 45% HFD combined with 10% high-fructose solution in drinking water. Mice received AAP via *ad libitum* dietary supplementation at doses of 50, 100, and 200 mg/kg/day. The experimental design detailing animal groupings and treatment parameters is documented in Supplementary Table S1, with the overall study timeline illustrated in Figure 1A.

### 2.3 Animal behavioral experiments

Voluntary activity of mice was assessed using an open-field test, while the Y-maze test was primarily utilized to evaluate rodents' spatial working memory (20). The Morris water maze test is a classic experiment used to assess learning and memory in rodents (21). Following acclimation training, positioning cruise experiments were performed four times daily over a 5-days period. The results of the positioning cruise experiments on Days 1, 3, and 5 were recorded. In these experiments mice were placed into a pool at four random entry points facing the pool wall, and the total distance traveled by the mice and the time required after entering the water to find a hidden platform and stand on it (i.e., evasion latency) were recorded automatically using the Super Maze Animal Behavioral Trajectory Analysis System. On the second day after the localization cruise experiment, the platform was removed and the spatial exploration experiment was carried out. Over a 60 s interval, the frequency of mouse crossings over the original platform was documented, while the distance traveled within the target quadrant and the total distance covered were systematically quantified.

### 2.4 Serum metabolite extraction and detection

At the end of the experiment, the mice were then placed in an induction chamber and exposed to 4% isoflurane-oxygen mixture until they were unconscious, as confirmed by the lack of a pedal withdrawal reflex. Blood was collected from the eyeballs of anesthetized mice and centrifuged at 1,000 × g for 20 min to separate the supernatant, resulting in serum. Serum metabolic analyses in mice were conducted following established laboratory protocols (22). Substance annotation was performed by matching the self-built secondary mass spectrometry database BiotreeDB (V2.1). Combined with multivariate statistical analysis, the original data were converted to mzXML format using ProteoWizard software to screen metabolic markers.

### 2.5 Histopathological and morphological observations

Paraffin sections of brain and colon tissues were subjected to xylene and gradient ethanol for dewaxing and rehydration. After washing with phosphate buffered solution (0.1 mol/L pH 7.4), nuclei were stained with hematoxylin for 6 min, followed by 1% hydrochloric acid alcohol differentiation, indirect rinsing in tap water for 20 min, counterblueing with 1% dilute ammonia, cytoplasm staining with eosin for 3 min, conventional ethanol dehydration, and xylene exposure for clarification. The slices were hermetically sealed using a neutral resin and then imaged using a light microscope after being allowed to dry in the air.

### 2.6 RT-qPCR

According to the reference (23), RNAiso Plus (Total RNA Extraction Reagent) kits were utilized to extract RNA from the colon tissues and brain of mice. Reverse transcribe RNA to cDNA following the instructions provided with the reverse transcription kit (RT mix with DNase, All-in-One). Gene mRNA levels were detected by real-time quantitative PCR using a Light Cycler 480 Real-Time System (EMCLAB, Germany). Mouse-specific primers are listed in Table 1.

**Table 1:** Primer sequences used for semi-quantitative RT-PCR analysis.

**Forward primer**		**Reverse primer**
*Snap-25*	CTGGCTGATGAGTCCCTGG	GACCGACTACTCAGGGACC
*Psd-95*	TCTGTGCGAGAGGTAGCAGA	AAGCACTCCGTGAACTCCTG
*Bace-1*	CCGGCGGGAGTGGTATTATGAAGT	GATGGTGATGCGGAAGGACTGATT
*Rage*	TATGGGGAGCTGTAGCTGGT	CAGAGCCTGTGACCCTGATG
*Il-1β*	TGACGGACCCCAAAAGATGA	TCTCCACAGCCACAATGAGT
*Tnf-α*	CCCTCACACTCAGATCATCTTCT	GCTACGACGTGGGCTACAG
*Occludin*	ACGGACCCTGACCACTATGA	TCAGCAGCAGCCATGTACTC
*Zo-1*	ACCCGAAACTGATGCTGTGGATAG	AAATGGCCGGGCAGAACTTGTGTA
*Muc-2*	AGGGCTCGGAACTCCAGAAA	CCAGGGAATCGGTAGACATCG
*Il-6*	TTCCATCCAGTTGCCTTCTTG	TATCCTCTGTGAAGTCTCCTCTC
*Il-10*	GCTCCAAGACCAAGGTGTCTACAA	CCGTTAGCTAAGATCCCTGGATCA
*Gapdh*	TGGAGAAACCTGCCAAGTATGA	TGGAAGAATGGGAGTTGCTGT

### 2.7 Detection of amyloid content in brain tissue and oxidative stress in colonic tissues

β amyloid in mouse brain tissue was determined according to the instructions of Aβ Amyloid ELISA kit (Shanghai Xinle Biotechnology Co., Ltd., China). MDA content, CAT, SOD, and GSH activities in mouse colon tissues were detected using Nanjing Jiancheng Kit (Nanjing Jiancheng Bioengineering Institute, China).

### 2.8 Fecal 16s rRNA sequencing analyses

Fecal 16S rRNA sequencing analyses were conducted following established laboratory methods (22). BIOTREE was entrusted with conducting high-throughput sequencing and bioinformatic analysis targeting the hypervariable V3–V4 region of the 16S rRNA gene. Following sequencing completion, raw paired-end reads underwent rigorous processing through quality control and filtering procedures to obtain high-quality sequences. Subsequent bioinformatic processing involved clustering of Operational Taxonomic Units (OTUs) at 97% similarity threshold followed by taxonomic classification using reference databases. To elucidate β-diversity patterns among distinct sample groups, multivariate statistical approaches including Principal Coordinates Analysis (PCoA) and Principal Component Analysis (PCA) were employed for dimensionality reduction visualization. Differential analysis was performed using Student's t-test complemented by LEfSe (Linear Discriminant Analysis Effect Size) algorithm to identify statistically significant variations in microbial composition and community structure across experimental groups. Furthermore, bivariate correlation analysis (Spearman's rank correlation) was implemented to investigate potential associations between discriminant bacterial taxa and host physiological parameters, including serum metabolite profiles and relevant clinical indices.

### 2.9 Statistical analyses

All data are expressed as means ± SEM (*n* ≥ 6) and were analyzed by Tukey's one-way ANOVA using SPSS 19.0. *p* < 0.05 was considered to be statistically significant difference.

## 3 Results

### 3.1 Effects of AAP dietary supplementation on mice learning and memory impairments induced by HFFD

Animal behavioral experiments have gradually become the most commonly used models for evaluating animal learning and memory. Results from the open-field (Figure 1B) and Y-maze (Figures 1C, D) experiments showed no significant difference in total arm-in distance, total number of arm-ins, or percentage of alternations between ND and ND + AAP groups, that is, AAP did not significantly affect the mice's voluntary movement ability. Compared with the ND group, the overall distance traveled and the proportion of mice entering the three different arms consecutively were considerably lower in the HFFD group, indicating that motor and working memory abilities were notably impaired. The impairment was significantly alleviated by dietary supplementation with 100 and 200 mg/kg/day of AAP.

**Figure 2:**
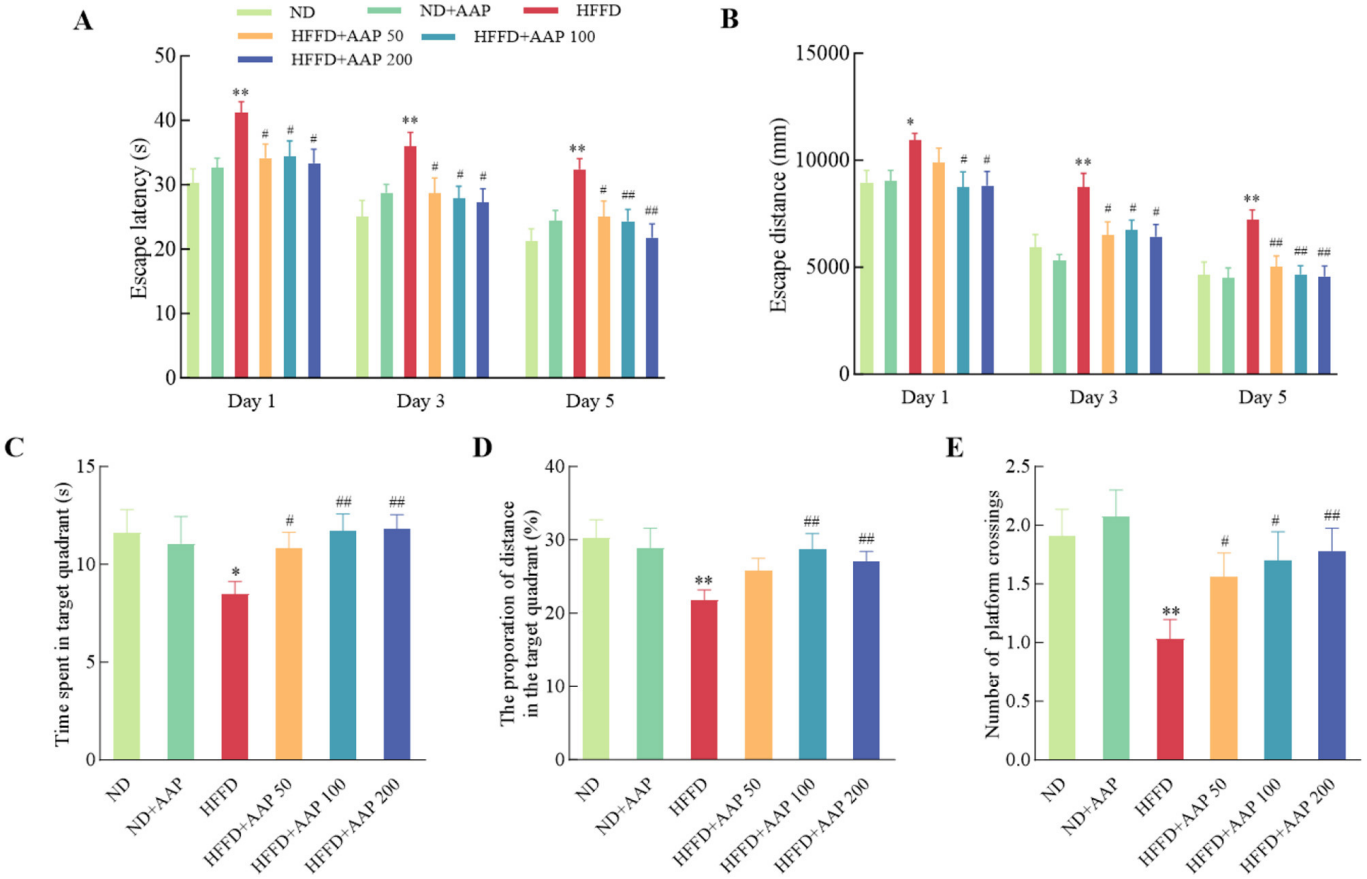
Suppression of HFFD-induced memory loss by AAP administration measured in a water maze test. Escape latency **(A)**, escape distance during the place navigation test **(B)**, time spent in the target quadrant **(C)**, percentage of total distance traveled within the target quadrant **(D)**, and number of platform crossings **(E)** during the probe trial were recorded. Data are presented as means ± SEM, *n* = 10. *p < 0.05, **p < 0.01, vs. ND group, ^#^*p* < 0.05, ^##^*p* < 0.01 vs. HFFD group.

To systematically investigate the therapeutic potential of AAP intervention for learning and cognitive impairments, the Morris Water Maze behavioral assay was employed in the present study (Figure 2), with particular focus on spatial memory acquisition and retention capacities. The mice in the HFFD group had a significantly longer escape latency compared to the ND group. It is worth noting that dietary supplementation with different concentrations of AAP effectively rescued this phenotype and reduced the platform-finding time. Figure 2B also showed a notable disparity in the evasion distances of the mice in each group. Again, AAP effectively reduced HFFD evasion distance. Figures 2C–E showed the results of spatial exploration experiments, in which the mice in the AAP intervention group displayed a significantly increased number of times they traversed their platforms, increased time spent in the quadrant containing the platforms, and increased distance traveled, i.e., AAP effectively ameliorated mice learning and cognitive deficits induced by HFFD.

### 3.2 Effects of AAP intervention on HFFD-induced neuronal damage and inflammatory responses in the mouse brain

**Figure 3:**
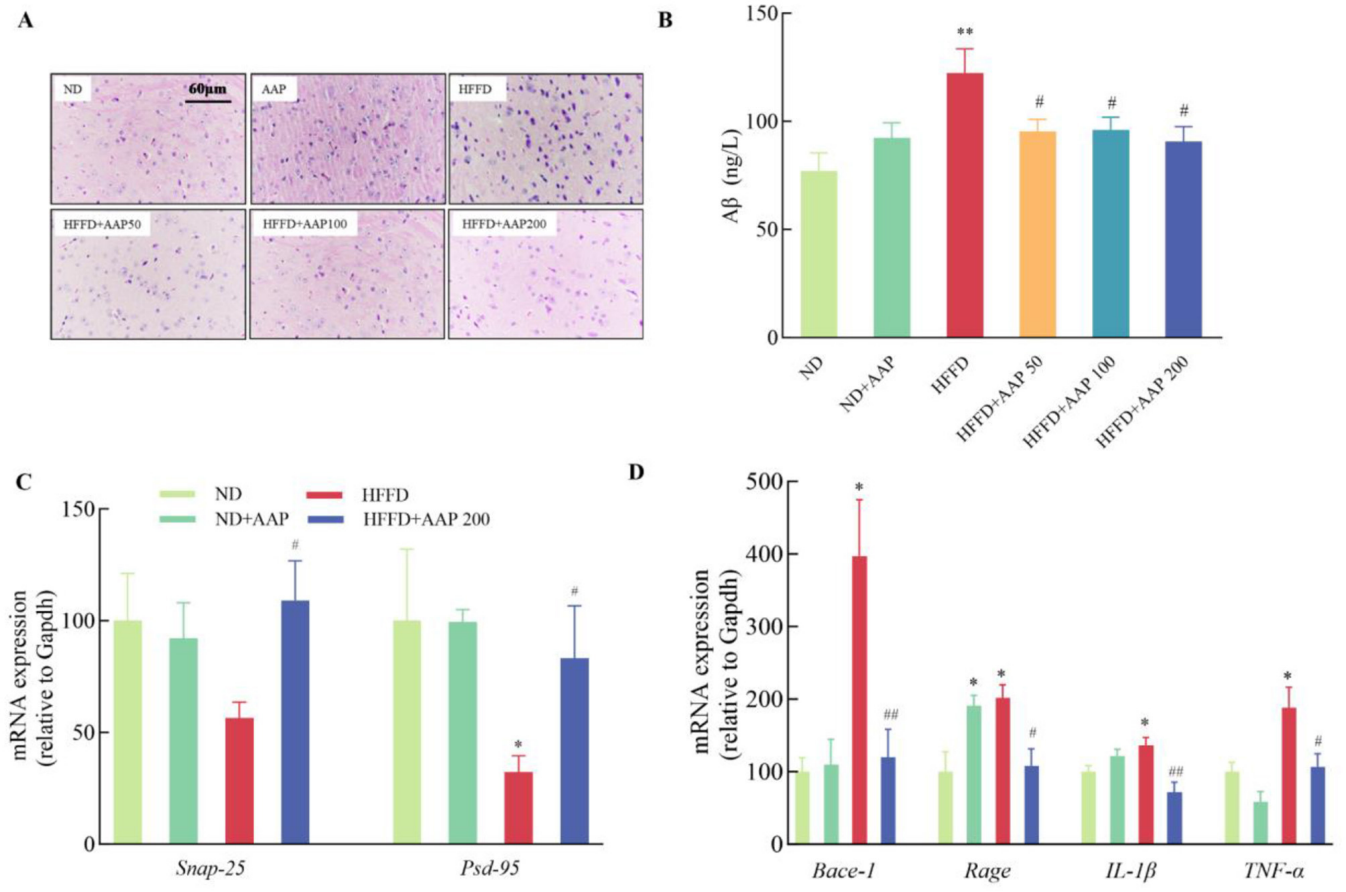
Effects of AAP on HFFD-induced neuronal damage and inflammatory responses in mouse brain. **(A)** H&E staining of mouse cerebral cortex (400×); **(B)** Aβ amyloid content; mRNA expression levels of genes related to **(C)** synaptic plasticity; **(D)** Aβ amyloid synthesis and inflammatory response in the mouse brain. Data are presented as means ± SEM, *p < 0.05, **p < 0.01, vs. ND group, ^#^*p* < 0.05, ^##^*p* < 0.01 vs. HFFD group.

To study the effect of dietary supplementation with AAP on HFFD-induced neuronal damage, the HE staining method was applied to observe neuronal cell morphology in the cortical area of the mouse brain (Figure 3A). Compared with the ND group, the neuronal cells in the brains of HFFD-induced mice were arranged in a loose and disorganized manner, and the neurons vanished or suffered nuclear solidification morphological changes. AAP supplementation obviously rescued neuron morphological abnormalities in the cortices of HFFD-induced mice. Aβ amyloid deposition is a primary cause of Alzheimer's disease. To investigate whether AAP would reduce HFFD-induced Aβ amyloid deposition and improve learning memory function, Aβ expression in the cerebral cortex was measured by ELISA. Figure 3B ELISA results demonstrated that the content of Aβ amyloid deposits was dramatically higher in the HFFD group, and dietary AAP supplementation substantially reduced Aβ content in the brains of HFFD-fed mice by approximately 25.32%.

Furthermore, Real-time quantitative PCR results (Figure 3C) showed that supplementation with 50, 100 or 200 mg/kg body weight/day AAP significantly increased *Snap-25* and *Psd-95* mRNA levels and enhanced synaptic plasticity compared with the HFFD. For in-depth analysis of pathological mechanisms, the HFFP+APP 200 model was systematically selected based on dose-response characteristics. Remarkable downregulation of HFFD-induced mRNA expression was observed in AAP-treated specimens, specifically in β-site amyloid precursor protein cleaving enzyme-1 (*Bace-1*) and receptor for advanced glycation end products (*Rage*) transcripts (Figure 3D), with reduction rates of 69.77 ± 9.68% and 46.29 ± 11.59%, respectively (*p* < 0.05), establishing a molecular basis for attenuated Aβ deposition. Inflammation is also thought to be an important factor in Aβ plaque accumulation, and thus in disease. Glial cell overactivation will lead to release of large amounts of pro-inflammatory factors (13). As shown in Figure 3D, AAP treatment significantly suppressed the mRNA expression of inflammatory mediators *Il-1*β and *Tnf-*α by 47.31 ± 10.24% and 43.41 ± 9.57%, respectively.

### 3.3 Equations effects of AAP on mice intestinal barrier damage induced by HFFD

**Figure 4:**
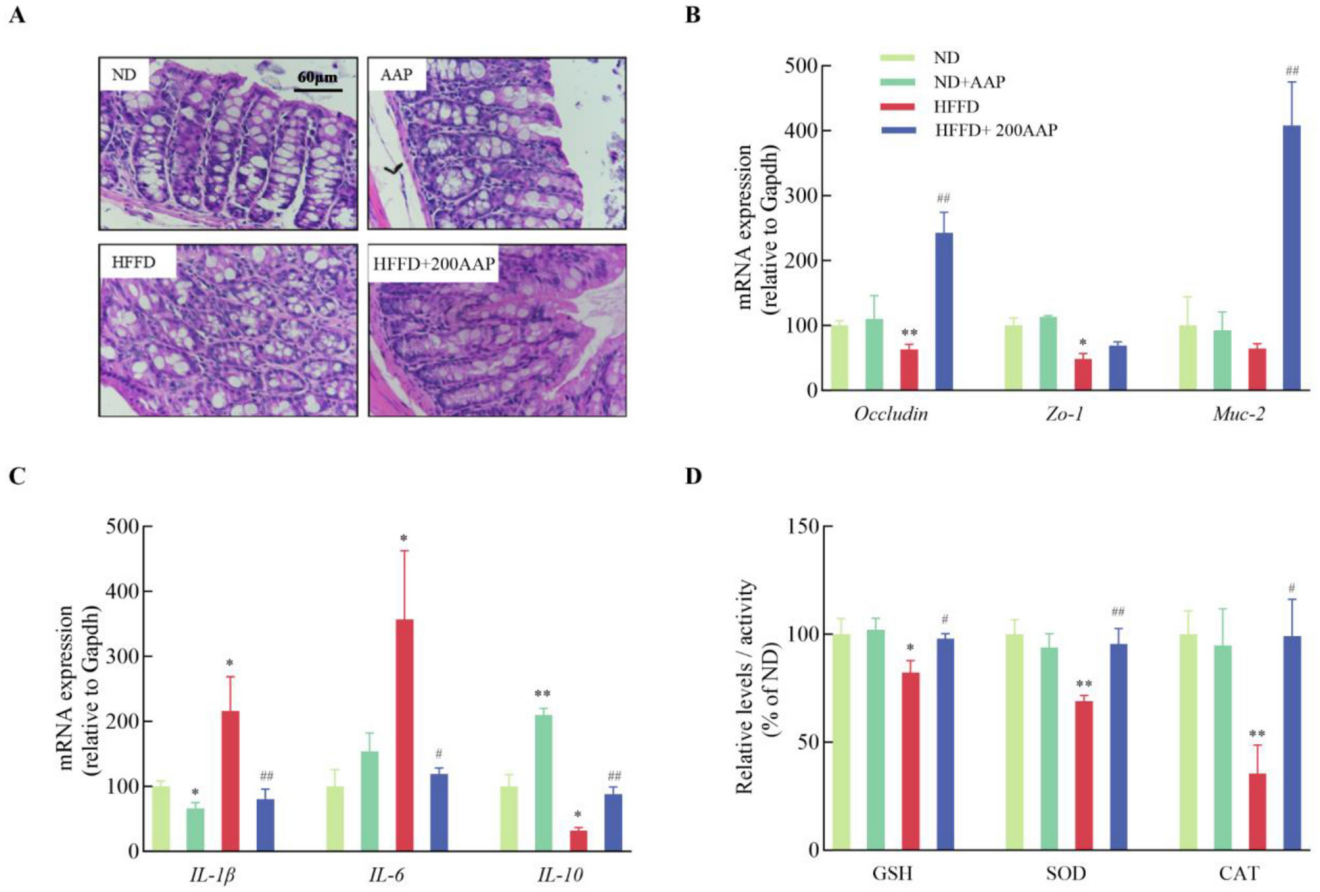
Effect of AAP on HFFD-induced intestinal barrier damage in mice. **(A)** H&E stained images of colon tissue morphology (400×); mRNA levels of **(B)** tight junction protein and **(C)** inflammation-related genes; **(D)** expressions of oxidative stress-related enzymes in mouse colon tissue. Data are presented as means ± SEM, *p < 0.05, **p < 0.01, vs. ND group, ^#^*p* < 0.05, ^##^*p* < 0.01 vs. HFFD group.

Intestinal barrier damage causes changes in intestinal permeability, inducing a series of related tissue injuries caused by inflammatory responses, and aggravating disease development (24). In contrast to the ND group, colon morphology and structure in HFFD group mice showed varied microvillus lengths accompanied by rupture of villi, nuclear solidification, disrupted tight junctions, and disorganized cell morphology. Dietary supplementation with AAP improved the quality of tight junctions and morphology of microvilli (Figure 4A). These results indicated that AAP intervention reduced histomorphological changes in HFFD-exposed colonic tissues in mice. To investigate the ameliorative effect of dietary AAP supplementation on intestinal barrier damage, the mRNA levels of tight junction protein-related genes (*Occludin* and *Zo-1*) and mucosal protein-related genes (*Muc-2*) in the intestinal tissues of mice were detected using qRT-PCR (25). As demonstrated in Figure 4B, colonic tissues from HFFD + AAP-treated mice showed a 2.85 ± 0.31-fold increase in *Occludin* mRNA expression (*p* < 0.01) and a 5.34 ± 0.52-fold increase in *Muc-2* (*p* < 0.01) compared to the HFFD group.

Additionally, in the colonic tissues of mice in the HFFD group, the expression levels of *Il-1*β and *Il-6* mRNA were significantly elevated, while *Il-10* mRNA levels were markedly reduced compared to the ND group (Figure 4C). This suggests that HFFD exacerbates intestinal inflammatory responses, in agreement with previous research (26). On the contrary, dietary supplementation with AAP significantly ameliorated the inflammation. Antioxidant levels in intestinal tissues are shown in Figure 4D, which shows that the HFFD + AAP group significantly up-regulated CAT and SOD enzyme activities, and increased GSH content in mouse colon tissues compared with these levels in HFFD group mice. These results indicated that AAP exerts a significant ameliorating effect on the imbalance in the inflammatory response and oxidative stress state in the colon tissues of HFFD-treated mice.

### 3.4 Effects of AAP on HFFD-induced intestinal flora in mice

**Figure 5:**
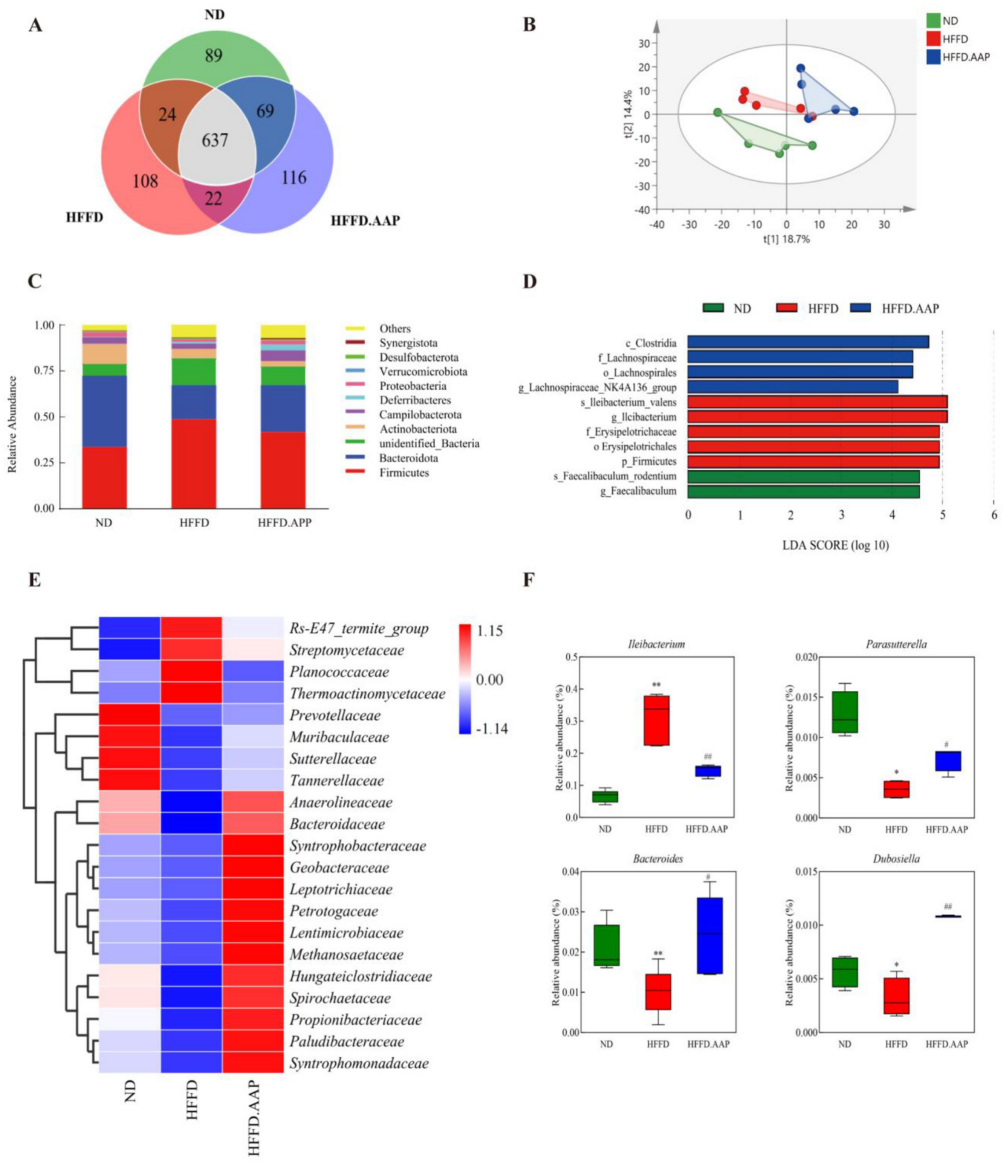
Effects of AAP on the intestinal microbiota of HFFD-induced mice. **(A)** Venn diagram; **(B)** PCoA; **(C)** Gut microbial structure at the phylum level; **(D)** Histogram of LDA discrimination; **(E)** Heat map of intestinal flora at the family level for each group of mice; **(F)** Abundance of differentially present bacteria. Data are presented as means ± SEM, *p < 0.05, **p < 0.01, vs. ND group, # *p* < 0.05, ## *p* < 0.01 vs. HFFD group.

The Venn diagrams in Figure 5A were employed to evaluate the similarity and overlap in OTU composition among the various groups. There are 89, 108, and 116 OTUs in ND, HFFD, and HFFD + AAP group separately. Shannon index is a measure of species richness and evenness, and Simpson index mainly reflects the evenness of the distribution of species in the community. Both Shannon index and Simpson index of the HFFD + AAP group were higher than those of the HFFD group, suggesting that the intestinal flora of the mice were more enriched and even after dietary supplementation with AAP (Supplementary Figure S3). Furthermore, PCoA at the OTU level demonstrated that all ND, HFFD, and HFFD + AAP samples each clustered together, and their structural profiles could be separated, indicating that the intestinal flora structure varied significantly among the groups and that dietary AAP supplementation induced a change in the β-diversity of intestinal microorganisms in mice on an HFFD (Figure 5B). At the phylum level, the microbial community structure of mouse feces is illustrated in Figure 5C, which was primarily composed of *Firmicutes, Bacteroidota, Actinobacteriota, Campilobacterota*, and *Deferribacteres*, consistent with the results of previous studies (27). AAP supplementation strikingly repressed the increase in *Firmicutes* abundance and the downregulation of *Bacteroidota* abundance induced by HFFD. It also significantly increased the abundance of *Deferribacteres*, a phylum of *Defertilobacteria*, which has been reported to correlate positively with memory, as compared with the HFFD group (28).

Due to differences in mouse fecal flora composition and structure across treatment groups and to understand information on species with significant differences, LEFSe used linear discriminant analysis (LDA) to identify specific altered bacterial phenotypes and key species with significant differences between groups. As shown in Figure 5D, LEFSe results showed that a total of 11 species information with significant differences between groups were found, of which the ND group had four categories of key species with significant differences, namely, *c_Clostridia, f_Lachnospiraceae, o_Lachnospirales*, and *g_Lachnospiraceae_NK4A136_group*; the HFFD had five categories of significantly different key species, *s_lleibacterium valens, g_lleibacterium, f_Erysipelotrichaceae, o_Erysipelotrichales* and *p_Firmicutes*; the HFFD + AAP group had two categories of significantly different key species, *s_Faecalibaculum_rodentium* and *g_Faecalibaculum*.

To systematically evaluate intergroup disparities in intestinal microbiota, fecal microbial composition was analyzed through hierarchical clustering visualization at the taxonomic family level. As illustrated in Figure 5E, AAP supplementation markedly boosted the relative abundance of *Syntrophomonadaceae, Paludibacteraceae, Propionibacteriaceae, Spirochaetaceae, Hungateiclostridiaceae*, and downregulated *Planococcaceae, Thermoactinomycetaceae, Streptomycetaceae, Rs-E47_termite_group* relative abundance compared to the HFFD model group. Strains with significant differences were identified using an intergroup t-test. As shown in Figure 5F, dietary AAP supplementation significantly modulated gut microbiota composition in comparison to the HFFD group, with a marked increase in the relative abundance of *Parasutterella* (1.09-fold, *p* < 0.05), *Bacteroides* (1.38-fold, *p* < 0.05), and *Dubosiella* (2.38-fold, *p* < 0.01), alongside a significant reduction in *Ileibacterium* levels (*p* < 0.01).

### 3.5 Changes in serum biomarkers in the HFFD-fed mice after AAP consumption

**Figure 6:**
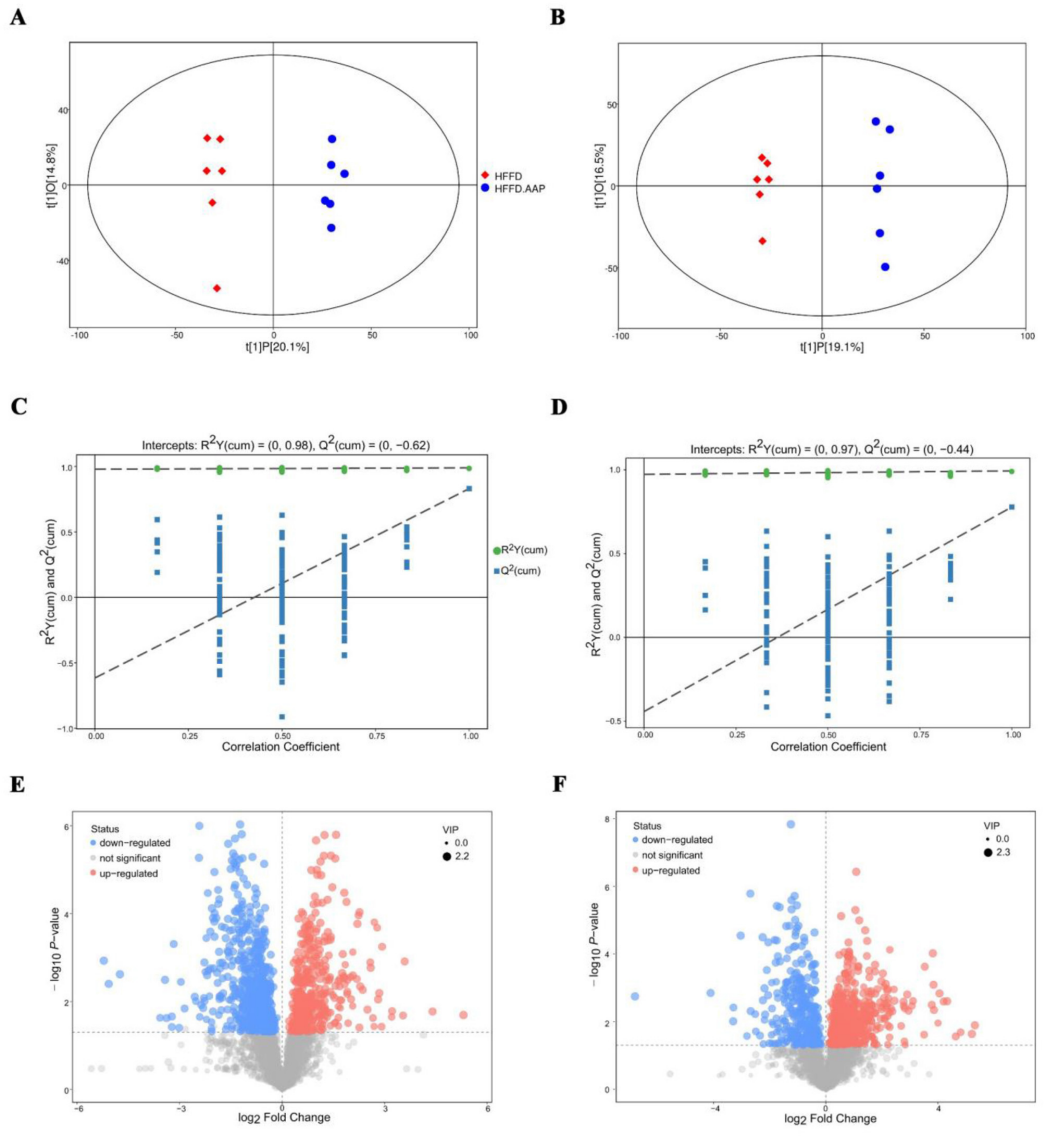
Biomarker identification in AAP treated mice using serum metabolomics. OPLS-DA score plot in **(A)** positive ion mode and **(B)** negative ion mode; OPLS-DA replacement test (200 times) in **(C)** positive ion mode and **(D)** negative ion mode; volcano map of differential metabolite screening in **(E)** positive ion mode and **(F)** negative ion mode.

The OPLS-DA score plot (Figures 6A, B) clearly demonstrates a highly significant differentiation between the serum samples of mice in the HFFD and HFFD + AAP groups. As illustrated in Figures 6C, D, the model exhibits good predictability without overfitting and can be analyzed in the next step. The differential metabolite screening results were visualized as volcano plots, shown in Figures 6E, F. Substances meeting the following requirements were identified as differential metabolites: (1) Variable Importance Projection (VIP) value >1; (2) Significant difference *p*-value < 0.01; and (3) Exclusion of heterologous metabolites in the context of the literature. A total of 81 differential metabolites with potential as biomarkers were identified in the serum of HFFD-fed mice after AAP consumption (Supplementary Table S2). Among these, metabolites 1–55 were acquired using positive ion mode, while metabolites 56–81 were acquired by negative ion mode scan. The compounds L-Proline, L-Lysine, Citrulline, L-Phenylalanine, (Â±)- Tryptophan, and L-Norleucine were detected in both positive and negative ionization modes.

### 3.6 Relevance analysis

**Figure 7:**
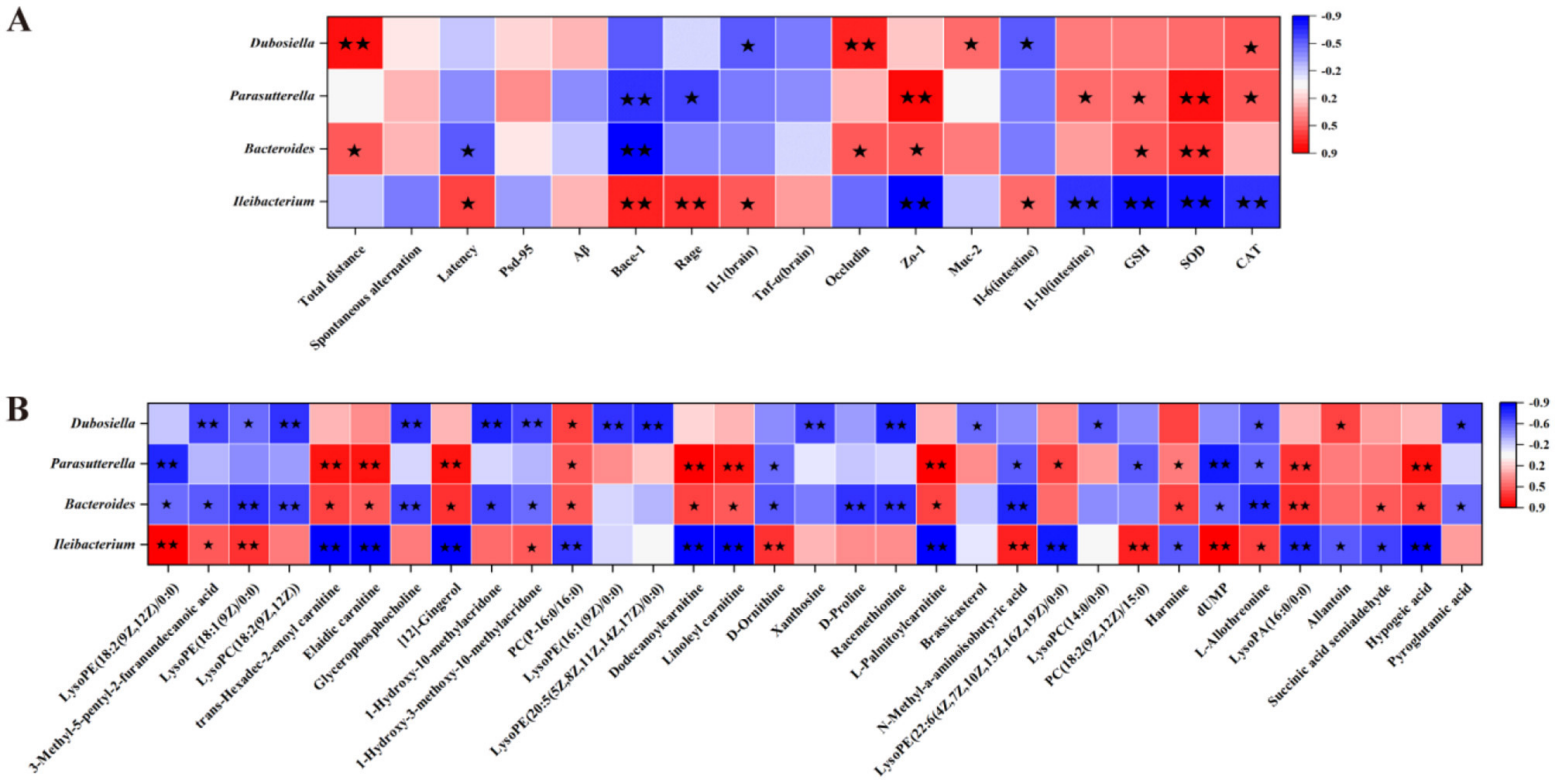
Correlation analysis. **(A)** Correlation analysis between characteristic bacteria and basic indices, and **(B)** Correlation analysis between characteristic bacteria and differential serum metabolites. Deeper color represents greater correlation. ^⋆^ indicates *p* < 0.05, ^⋆⋆^ indicates *p* < 0.01.

To elucidate multidimensional interactions within the gut-brain axis, Spearman's rank correlation analysis was systematically conducted based on experimental datasets encompassing intestinal microbiota profiles, serum metabolomic signatures, and neurotrophic parameters. Figure 7A reveals significant positive correlations between *Dubosiella* abundance and both locomotor activity indices (total distance) and intestinal barrier integrity markers (*Occludin*). Bacteroides demonstrated synergistic associations with antioxidant enzymes (SOD, GSH) and tight junction proteins (*Zo-1*), whereas inverse correlations were observed with *Bace-1* expression levels. There was a strong negative correlation with *Bace-1* and a notably positive correlation with SOD and *Zo-1* for *Parasutterella*. *Ileibacterium* exhibited a significant positive association with *Bace-1* and *Rage*, and significant negative correlations with SOD, *Il-10*, CAT, GSH, and *Zo-1*. Metabolite levels with *p*-values ≤ 0.001 were heat mapped against the abundance of colonies that showed a significant difference in presence. The results showed that significantly different flora abundances correlated with serum metabolites, the intestinal flora may regulate body metabolism by regulating flora metabolites (Figure 7B).

## 4 Discussion

A strong relationship between diet and risk factors for neurodegenerative disorders has been demonstrated. Excessive saturated fatty acid intake enhances neurodegeneration in Alzheimer's disease (AD) and Parkinson's disease (PD) by exacerbating oxidative stress and lipid peroxidation, and high-calorie intake is positively associated with early onset of Hyperactivity disorder (HD) (29). Increased saturated fat intake can also cause an inflammatory response, which can lead to peripheral immune cell entry into the central nervous system (30), which may be an exacerbating factor in neurological disease symptoms pursuant to poor dietary intake. Numerous studies have found that dietary intake of nutrients and their metabolites can regulate neuroinflammation and exert beneficial effects on neurological functions and metabolic disorders (31). The United Nations has recommended “one meat, one vegetable, and one mushroom” as the dietary pattern for the twenty-first century (32, 33). *Hericium, Ganoderma, Lentinula*, and *Psilocöm* are a few of the mushrooms that can be used to improve brain health and treat cognitive decline. Proactive substances such as cordycepin from *Cordyceps*, lentinan from *Lentinula*, and hericenones from *mushrooms* and mycelia of *lion mane* can reduce stress, improve sleep quality, and improve memory by stimulating the brain and reducing cortical neuron shrinkage (34–36). In the current investigation, it was discovered that learning and memory in mice were markedly enhanced by active polysaccharides isolated from *Auricularia Auricula-judae*, and that prolongation of escape latency and reduction in the percentage of total distance traveled within the target quadrant in the water maze induced by a high-caloric-energy diet were significantly inhibited.

The gut, as the body's largest immunological organ, and the microorganisms that live there are essential for regulating metabolism and endocrinology. Macrogenomic data suggest that extrinsic factors, including diet, greatly outweigh the contribution of genetic factors in regulating the structure and function of the intestinal flora (37). Customized diets have the potential to alter an organism's internal metabolism, which can have an impact on health (38, 39). Animal experiments and population studies have shown that changes in microbiota composition in response to a hypercaloric diet include an increase in the proportion of *thick-walled bacilli* and *mycobacteriophages*, and increased abundance of *Dentinobacterium, Bacillus*, and *Clostridium* (all of which belong to the thick-walled bacilli phylum). Compared to animal-based diets, plant-based diets promote gut microbiota complexity, increase the abundance of dietary fiber-fermenting microbiota, and induce increases in metabolites including SCFA in the gut and circulating blood (40). Zhang et al. conducted a comparative study on the regulatory effects of *Auricularia auricula* and AAP on the intestinal microbiota of rats with hyperlipidemia. They found that AAP strongly stimulated certain SCFA-producing bacteria with lower abundance, such as *Flavonifractor* and *Clostridium IV* (41). Our previous study found that supplementation with AAP (from Qinba mountains) significantly changed mouse intestinal flora composition compared to the normal C57BL/6J mouse group. The relative abundances of *Lactobacillus johnsonii, Weissella cibaria, Kosakonia cowanii, Enterococcus faecalis, Bifidobacterium animalis*, and *Bacteroides uniformis* were remarkably enhanced, whereas *Firmicutes bacterium M10-2* was downregulated (22). In the present study, AAP supplementation significantly boosted the abundance of *Dubosiella* in the intestinal tract of HFFD mice, upregulated the abundance of *Bacteroides* and *Parasutterella*, and markedly reduced the abundance of *Ileibacterium* to levels similar to those in normal C57BL/6J mice (Figure 5F).

It has been found that *Dubosiella* can exert an inhibitory effect on AD development through the synthesis of palmitoleic acid (42). In addition, *Dubosiella* can regulate the homeostasis of intestinal flora, e.g., SCFAs produced by *Dubosiella newyorkensis* can regulate intestinal immune homeostasis by activating the AhR-IDO1-Kyn metabolic circuit (43). *Bacteroides* has a wide range of health benefits and is one of the most abundant genera in the human gut flora. Previous studies have shown that *Bacteroides uniformis* improves locomotor performance in mice and humans (44). Key features associated with Aβ loading include a reduction in *Bacteroides*, while the addition of *Bacteroides ovatus* was effective in improving AD symptoms (45). Additionally, Autism spectrum disorder (ASD)-like behaviors can be improved by regulating intestinal amino acid transport protein levels and serum glutamine levels through supplementation with *Bacteroides uniformis* (46). *Bacteroides* possesses 20% of the genome to regulate the degradation of polysaccharides, e.g., it can degrade complex arabinoxylans by manipulating the polysaccharide-utilization loci while releasing ferulic acid, which in turn exerts immunomodulatory effects (47, 48). *Parasutterell* is associated with a high calorie diet-induced inflammation of the hypothalamus, which controls appetite by regulating the secretion of multiple hormones, and dysregulation of appetite and satiety can lead to weight gain (49). Furthermore, its ability to regulate the process of bile acid synthesis by altering bile acid metabolites has a potential therapeutic effect on hypercaloric diets (50). Correlation analysis is the starting point for gut-brain axis research. However, correlation analyses have limitations, including the inability to target specific strains of bacteria and the inability to determine whether gut-brain interactions show a threshold effect or a dynamic equilibrium (e.g., a critical abundance of a particular group of bacteria is required to affect neurotransmitters). These limitations need to be compensated for by causal inference experiments, refined study design and integration of multidisciplinary approaches (e.g., fecal microbiota transplantation assays, targeted colony editing, etc.). Further verification is still needed to prove that the characteristic bacteria in the mice intestine play a key role in cognitive intervention after dietary supplementation with AAP.

A lot of studies have demonstrated that the intestinal flora can achieve bidirectional information exchange with the central and enteric nervous systems via direct and indirect pathways involving multiple systems, including neural, endocrine, and immune systems, forming the flora-gut-brain axis (51). Intestinal flora effects on the nervous system include direct interactions with enterocytes and enteric neurons, metabolite-mediated inflammation, vagal activation, HPA axis modulation, and immune system regulation (52–54). Ma et al. demonstrated that *Lactobacillus, Akkermansia, Oscillospira*, etc. were associated with cognitive decline by fecal microbiota transplantation (55). *Akkermansia* has also been shown in earlier studies to be a beneficial bacterium that improves cognitive deficits (56). Current research suggests that gut flora has a causal effect on cognitive function through metabolites, immune modulation and gut-brain axis signaling. However, this conclusion is mainly based on animal model intervention experiments (flora transplantation, direct intervention with metabolites) and human intervention studies (probiotic/prebiotic trials, dietary interventions). Gut microecological changes induced by high-fat, high-sugar diets are important factors in the development of cognitive dysfunction and anxiety-like behaviors. Non-digestible polysaccharides cannot be digested or utilized by the body. One of their key biological roles is to maintain intestinal flora homeostasis (57). Polysaccharides improve intestinal integrity; alleviate intestinal mucosal damage; increase the activity of carbohydrate-active enzymes; promote SCFAs production; decrease endotoxin levels; downregulate inflammatory factor expression; upregulate tight junction protein expression; and regulate microglial cell maturation (58). Although structure-function relationships have not been clarified, some structural properties of these polysaccharides, including molecular weight, composition of monosaccharides, and the way their molecular chains are linked, may be crucial for their brain nutritional properties (59). The study demonstrated that AAP from the Qinba Mountains exerted a significant modulatory effect on serum metabolite profiles in mice fed a hypercaloric diet, with the differentiated genes mainly enriched in the glycerophosphate metabolism pathway. AAP up-regulated synaptic plasticity-related genes expressions, such as *Snap-25* and *Psd-95*, and suppressed *Il-1*β and *Tnf-*α inflammatory mediators levels, effectively ameliorating high-caloric diet-induced cognitive decline in mice. This polysaccharide was found to be primarily composed of mannose (>60%) and glucuronic acid in our earlier investigation (60). In a 5xFAD transgenic AD mice model, LIU et al. discovered that an 8-week therapy with Mannan oligosaccharide (MOS, 0.12% w/v) dramatically enhanced cognitive performance and spatial memory, along with decreased anxiety- and obsessive-like behaviors. Furthermore, MOS improved HPA axis disorders by upregulating norepinephrine expression and lowering corticosterone and corticotropin-releasing hormone levels (61). To what extent the brain health-promoting effects of AAP are dependent on their mannose content, their effector molecules, and the relationship between fine structure and function needs to be further investigated. This study demonstrates the potential of edible mushroom polysaccharides in improving cognitive function based on animal models; however, human clinical trials have not yet been conducted. The effective dosage, feasibility of translating mechanisms of action to humans, and long-term safety profiles still require systematic validation.

## 5 Conclusion

The therapeutic promise of AAP in nutritional interventions has been documented, whereas the mechanistic connections between its bioactive properties and high calorie diets-induced cognitive deterioration require further elucidation. In the present investigation, the capacity of AAP to mitigate HFFD-associated cognitive dysfunction was mechanistically linked to its coordinated regulation of intestinal microbiota composition and serum metabolic signatures in murine models. These results advance the current understanding of plant-derived polysaccharides as multifunctional modulators of the microbiota-metabolite axis, establishing AAP as a candidate for targeted dietary strategies against neurocognitive complications. Additionally, the mechanistic framework delineated herein supports the rational design of nutritional guidelines to counteract health risks associated with excessive caloric intake.

## Data Availability

The original contributions presented in the study are included in the article/Supplementary material, further inquiries can be directed to the corresponding author.
