# Supplementary material for: Ameliorating potential of Auricularia auricula-judae polysaccharides in mitigating hypercaloric diet-induced behavioral disorders through gut microbiota regulation
Source: Front Nutr. 2025 May 29;12:1585778. doi: 10.3389/fnut.2025.1585778 (PMC12158723; doi:10.3389/fnut.2025.1585778)
Supplement: Supplementary file 1 [file Table_1.docx]

Supplementary Material

# Supplementary Table 1 Animal experimental design

7-week-old SPF C57BL/6J male mice were purchased from Xi'an Jiaotong University (Shaanxi, China). SCXK 2018-001. Our animal experiment protocols were approved by the Animal Ethics Committee of the Laboratory Animal Centre of Northwestern University (Approval Code: NWU-AWC-20200401M) and carried out by the “Administrative Regulations on Laboratory Animals” of the National Science and Technology Commission of the People’s Republic of China. The mice were reared in a standard conditioned animal facility (relative humidity: 40±10%, temperature: 25±2°C, light/dark cycle: 12/12 h, clean bedding, free access to food and water). A standard diet (AIN-93M) and a 45% high-fat diet (TP230100) were purchased from TROPHIC Animal Feed High-Tech Co., Ltd. Nantong, China, and stored in a refrigerator at 4°C. The energy content of the standard diet was 3.6 kcal/g and that of the 45% high-fat diet was 4.5 kcal/g. Following one week of acclimatization feeding, the animals were grouped. To ensure a consistent baseline across groups, the body weight of each mouse was measured and used as a stratification variable. Stratified randomization was implemented according to the following protocol: mice with body weights within a ±2 g range were divided into blocks based on weight categories.Within each block, subjects were systematically assigned to one control group and five experimental groups (six groups total, n=10 per group) using a computer-generated random sequence produced by the Excel RAND ( ) function. The specific treatments were as follows: mice in the ND group were given AIN93M standard feed and distilled water; mice in the HFFD model group were fed the 45% high-fat feed and 10% high-fructose drinking water; mice in the ND+AAP negative control group were given standard chow containing 200 mg/kg/day of AAP and distilled water; and mice in the HFFD+AAP intervention groups were given 45% high-fat feed with 10% high-fructose drinking water containing 50, 100, or 200 mg/kg/day of AAP.

| Groups | Treatment |
| --- | --- |
| ND group (normal diet) | standard chow and distilled water |
| HFFD group (high-fat and high-fructose diets) | 45% high-fat feed and 10% high-fructose drinking water |
| ND+AAP group | standard chow containing 200 mg/kg/day of  AAP and distilled water |
| HFFD+AAP group | 45% high-fat diet supplemented with AAP at 50, 100, or 200 mg/kg/day, paired with 10% fructose drinking water. |

# Supplementary Table 2 Identification of serum metabolites in HFFD-induced mice treated with AAP

| No. | Metabolites | Sub Class | VIP value | P-value | experimental m/z | Molecular formula | HMDB ID | KEGG ID | HFFD+AAP vs. HFFD |
| --- | --- | --- | --- | --- | --- | --- | --- | --- | --- |
| POS |  |  |  |  |  |  |  |  |  |
| 1 | LysoPE(18:2(9Z,12Z)/0:0) | Glycerophosphoethanolamines | 2.167117435 | 0.000125579 | 478.2922977 | C_23_H_44_NO_7_P | HMDB0011507 | Not Available | ↓ |
| 2 | c8:1(9Z)/0:0) | Glycerophosphoethanolamines | 2.152630504 | 9.33056E-07 | 480.3091467 | C_23_H_46_NO_7_P | HMDB0011506 | Not Available | ↓ |
| 3 | LysoPE(18:3(6Z,9Z,12Z)/0:0) | Glycerophosphoethanolamines | 2.048775508 | 0.002384675 | 476.2753058 | C_23_H_42_NO_7_P | HMDB0011508 | Not Available | ↓ |
| 4 | LysoPE(16:1(9Z)/0:0) | Glycerophosphoethanolamines | 2.015857672 | 0.000237811 | 452.2775043 | C_21_H_42_NO_7_P | HMDB0011504 | Not Available | ↓ |
| 5 | LysoPE(20:5(5Z,8Z,11Z,14Z,17Z)/0:0) | Glycerophosphoethanolamines | 2.006991435 | 7.48079E-05 | 500.2723294 | C_25_H_42_NO_7_P | HMDB0011519 | Not Available | ↓ |
| 6 | LysoPE(22:6(4Z,7Z,10Z,13Z,16Z,19Z)/0:0) | Glycerophosphoethanolamines | 1.875618735 | 0.000878917 | 526.289328 | C_27_H_44_NO_7_P | HMDB0011526 | Not Available | ↑ |
| 7 | LysoPC(18:2(9Z,12Z)) | Glycerophosphocholines | 2.13703692 | 1.55813E-06 | 520.3385441 | C_26_H_50_NO_7_P | HMDB0010386 | Not Available | ↓ |
| 8 | Glycerophosphocholine | Glycerophosphocholines | 2.114910722 | 7.75123E-06 | 258.1092654 | C_8_H_20_NO_6_P | HMDB0000086 | C00670 | ↓ |
| 9 | PC(P-16:0/16:0) | Glycerophosphocholines | 2.066607465 | 0.000162533 | 718.5760885 | C_40_H_80_NO_7_P | HMDB0011206 | Not Available | ↑ |
| 10 | LysoPC(14:0/0:0) | Glycerophosphocholines | 1.860582372 | 0.000930143 | 468.3072553 | C_22_H_46_NO_7_P | HMDB0010379 | Not Available | ↓ |
| 11 | PC(18:2(9Z,12Z)/15:0) | Glycerophosphocholines | 1.784480011 | 0.000324082 | 744.5567312 | C_41_H_78_NO_8_P | HMDB0008132 | Not Available | ↓ |
| 12 | PC(18:2(9Z,12Z)/18:0) | Glycerophosphocholines | 1.749610404 | 0.001609784 | 786.5972435 | C_44_H_84_NO_8_P | HMDB0008135 | Not Available | ↓ |
| 13 | LysoPC(16:1(9Z)/0:0) | Glycerophosphocholines | 1.745231151 | 0.003426723 | 494.3252441 | C_24_H_48_NO_7_P | HMDB0010383 | C04230 | ↓ |
| 14 | LysoPC(22:4(7Z,10Z,13Z,16Z)) | Glycerophosphocholines | 1.635244244 | 0.006533922 | 572.3690005 | C_30_H_54_NO_7_P | HMDB0010401 | C04230 | ↓ |
| 15 | 3-Methyl-5-pentyl-2-furanundecanoic acid | Fatty acids and conjugates | 2.160038567 | 0.000150834 | 337.272568 | C_21_H_36_O_3_ | HMDB0031005 | Not Available | ↓ |
| 16 | trans-Hexadec-2-enoyl carnitine | Fatty acid esters | 2.12966225 | 5.55436E-06 | 398.3261291 | C_23_H_43_NO_4_ | HMDB0006317 | Not Available | ↑ |
| 17 | Elaidic carnitine | Fatty acid esters | 2.122672657 | 1.3077E-05 | 426.3572297 | C_25_H_47_NO_4_ | HMDB0006464 | Not Available | ↑ |
| 18 | Dodecanoylcarnitine | Fatty acid esters | 2.006536004 | 0.000176828 | 344.2783663 | C_19_H_38_NO_4_ | HMDB0002250 | Not Available | ↑ |
| 19 | Linoleyl carnitine | Fatty acid esters | 1.985994674 | 0.000148755 | 424.3416911 | C_25_H_46_NO_4_ | HMDB0006469 | Not Available | ↑ |
| 20 | L-Palmitoylcarnitine | Fatty acid esters | 1.943677592 | 0.000640006 | 400.3406088 | C_23_H_45_NO_4_ | HMDB0240774 | Not Available | ↑ |
| 21 | 2-Methylbutyroylcarnitine | Fatty acid esters | 1.789477291 | 0.007700892 | 246.1692747 | C_12_H_23_NO_4_ | HMDB0000378 | Not Available | ↓ |
| 22 | L-Hexanoylcarnitine | Fatty acid esters | 1.684779078 | 0.008997624 | 260.1848201 | C_13_H_26_NO_4_ | HMDB0000756 | Not Available | ↑ |
| 23 | Butyrylcarnitine | Fatty acid esters | 1.658271257 | 0.008418808 | 232.1535328 | C_11_H_21_NO_4_ | HMDB0002013 | C02862 | ↓ |
| 24 | [12]-Gingerol | Methoxyphenols | 2.099639093 | 5.66966E-05 | 396.3103219 | C_23_H_38_O_4_ | HMDB0036356 | Not Available | ↑ |
| 25 | 1-Hydroxy-10-methylacridone | Benzoquinolines | 2.093172532 | 1.14148E-05 | 226.0832208 | C_14_H_11_NO_2_ | HMDB0032983 | Not Available | ↓ |
| 26 | 1-Hydroxy-3-methoxy-10-methylacridone | Benzoquinolines | 2.082195503 | 5.13222E-06 | 256.0936273 | C_15_H_13_NO_3_ | HMDB0029322 | Not Available | ↓ |
| 27 | SM(d18:0/18:1(9Z)) | Phosphosphingolipids | 2.023438528 | 0.008230578 | 731.6037354 | C_41_H_83_N_2_O_6_P | HMDB0012089 | C00550 | ↑ |
| 28 | SM(d17:1/24:1(15Z)) | Phosphosphingolipids | 2.001932173 | 0.003204261 | 799.667732 | C_46_H_91_N_2_O_6_P | HMDB0011696 | Not Available | ↑ |
| 29 | SM(d18:1/16:0) | Phosphosphingolipids | 1.758862309 | 0.006881028 | 703.5735682 | C_39_H_79_N_2_O_6_P | HMDB0010169 | Not Available | ↑ |
| 30 | SM(d18:1/18:1(9Z)) | Phosphosphingolipids | 1.202915651 | 0.002732228 | 729.5885483 | C41H81N2O6P | HMDB0012101 | Not Available | ↑ |
| 31 | D-Ornithine | Amino acids, peptides, and analogues | 1.984228299 | 0.00022325 | 133.0969256 | C_5_H_12_N_2_O_2_ | HMDB0003374 | C00515 | ↓ |
| 32 | D-Proline | Amino acids, peptides, and analogues | 1.949893011 | 0.000422887 | 116.0707167 | C_5_H_9_NO_2_ | HMDB0003411 | C00763 | ↓ |
| 33 | Racemethionine | Amino acids, peptides, and analogues | 1.945551124 | 0.003837337 | 150.0580294 | C_5_H_11_NO_2_S | HMDB0033951 | C01733 | ↓ |
| 34 | N-Methyl-a-aminoisobutyric acid | Amino acids, peptides, and analogues | 1.903093042 | 0.00091369 | 118.0863371 | C_5_H_11_NO_2_ | HMDB0002141 | Not Available | ↓ |
| 35 | L-Proline | Amino acids, peptides, and analogues | 1.8238245 | 0.008451073 | 116.0707308 | C_5_H_9_NO_2_ | HMDB0000162 | C00148 | ↓ |
| 36 | L-Phenylalanine | Amino acids, peptides, and analogues | 1.782921463 | 0.003311362 | 166.0858554 | C_9_H_11_NO_2_ | HMDB0000159 | C00079 | ↓ |
| 37 | Citrulline | Amino acids, peptides, and analogues | 1.728275767 | 0.004954237 | 176.1025471 | C_6_H_13_N_3_O_3_ | HMDB0000904 | C00327 | ↓ |
| 38 | L-Norleucine | Amino acids, peptides, and analogues | 1.720927624 | 0.005167479 | 132.1017576 | C_6_H_13_NO_2_ | HMDB0001645 | C01933 | ↓ |
| 39 | 3,3,5-triiodo-L-thyronine-beta-D-glucuronoside | Amino acids, peptides, and analogues | 1.708353839 | 0.004616494 | 132.10169 | C_21_H_20_I_3_NO_1_0 | HMDB0010346 |  | ↓ |
| 40 | L-Lysine | Amino acids, peptides, and analogues | 1.686206768 | 0.005859513 | 147.1125228 | C_6_H_14_N_2_O_2_ | HMDB0000182 | C00047 | ↓ |
| 41 | L-Tyrosine | Amino acids, peptides, and analogues | 1.659814937 | 0.009612088 | 182.0808025 | C_9_H_11_NO_3_ | HMDB0000158 | C00082 | ↓ |
| 42 | Homocysteine thiolactone | Amino acids, peptides, and analogues | 1.608748358 | 0.009863923 | 118.0321619 | C_4_H_7_NOS | HMDB0002287 | Not Available | ↓ |
| 43 | Pipecolic acid | Amino acids, peptides, and analogues | 1.597560309 | 0.005890913 | 130.0860476 | C_6_H_11_NO_2_ | HMDB0000070 | C00408 | ↓ |
| 44 | Xanthosine | Purine nucleosides （Class） | 1.958215156 | 9.78139E-05 | 285.0820695 | C_10_H_12_N_4_O_6_ | HMDB0000299 | C01762 | ↓ |
| 45 | N-Palmitoylsphingosine | Ceramides | 1.957129012 | 0.003174836 | 538.5188348 | C_34_H_67_NO_3_ | HMDB0004949 | C00195 | ↓ |
| 46 | Cer(d18:0/16:0) | Ceramides | 1.658111133 | 0.007661173 | 540.5340148 | C_34_H_69_NO_3_ | HMDB0011760 | Not Available | ↓ |
| 47 | Brassicasterol | Ergostane steroids | 1.943086262 | 8.90874E-05 | 381.3496503 | C_28_H_46_O | HMDB0011181 | C08813 | ↓ |
| 48 | D-1-Piperideine-2-carboxylic acid | Hydropyridines | 1.773419696 | 0.004198333 | 128.0706728 | C_6_H_9_NO_2_ | HMDB0001084 | C04092 | ↓ |
| 49 | Deoxycytidine | Pyrimidine 2'-deoxyribonucleosides | 1.771229236 | 0.00600542 | 228.0975679 | C_9_H_13_N_3_O_4_ | HMDB0000014 | C00881 | ↑ |
| 50 | 1-Methylnicotinamide | Pyridinecarboxylic acids and derivatives | 1.716630834 | 0.008225451 | 137.0707328 | C_7_H_9_N_2_O | HMDB0000699 | C02918 | ↑ |
| 51 | 7-Ketocholesterol | Cholestane steroids | 1.715074816 | 0.004885805 | 401.3401282 | C_27_H_44_O_2_ | HMDB0000501 | Not Available | ↓ |
| 52 | (Â±)-Tryptophan | Indolyl carboxylic acids and derivatives | 1.635947242 | 0.008043969 | 205.0965356 | C_11_H_12_N_2_O_2_ | HMDB0030396 | C00806 | ↓ |
| 53 | Furanone A | Furanones | 1.585768138 | 0.00738739 | 85.02882881 | C_4_H_4_O_2_ | HMDB0094691 | C17602 | ↓ |
| 54 | Harmine | Harmala alkaloids （Class） | 1.525888112 | 0.000156562 | 213.1018319 | C_13_H_12_N_2_O | HMDB0030311 | C06538 | ↑ |
| 55 | Persicaxanthin | Diterpenoids | 1.524205228 | 0.003855375 | 385.2729761 | C_25_H_36_O_3_ | HMDB0034952 | Not Available | ↑ |
| NEG |  |  |  |  |  |  |  |  |  |
| 56 | Traumatic acid | Fatty acids and conjugates | 1.975944709 | 0.005704265 | 227.1291326 | C_12_H_20_O_4_ | HMDB0000933 | C16308 | ↓ |
| 57 | Succinic acid semialdehyde | Fatty acids and conjugates | 1.970696218 | 0.000241039 | 101.023407 | C_4_H_6_O_3_ | HMDB0001259 | C00232 | ↑ |
| 58 | Hypogeic acid | Fatty acids and conjugates | 1.93176803 | 0.000650691 | 253.2177687 | C_16_H_30_O_2_ | HMDB0002186 | Not Available | ↑ |
| 59 | 2-Ethyl-2-Hydroxybutyric acid | Fatty acids and conjugates | 1.841975445 | 0.001384697 | 131.070426 | C_6_H_12_O_3_ | HMDB0001975 | Not Available | ↑ |
| 60 | Caprylic acid | Fatty acids and conjugates | 1.812728522 | 0.002982365 | 143.1067419 | C_8_H_16_O_2_ | HMDB0000482 | C06423 | ↑ |
| 61 | Palmitoleic acid | Fatty acids and conjugates | 1.797130282 | 0.003039528 | 253.2183515 | C_16_H_30_O_2_ | HMDB0003229 | C08362 | ↑ |
| 62 | Methylglutaric acid | Fatty acids and conjugates | 1.789580209 | 0.001840223 | 145.0496829 | C_6_H_10_O_4_ | HMDB0000752 | Not Available | ↑ |
| 63 | dUMP | Pyrimidine deoxyribonucleotides | 2.167650728 | 2.8638E-05 | 307.0319804 | C_9_H_13_N_2_O_8_P | HMDB0001409 | C00365 | ↓ |
| 64 | Racemethionine | Amino acids, peptides, and analogues | 2.110286825 | 0.001529642 | 148.0429311 | C_5_H_11_NO_2_S | HMDB0033951 | C01733 | ↓ |
| 65 | L-Allothreonine | Amino acids, peptides, and analogues | 2.062774337 | 4.52065E-05 | 118.0500363 | C_4_H_9_NO_3_ | HMDB0004041 | C05519 | ↓ |
| 66 | L-Proline | Amino acids, peptides, and analogues | 2.053123867 | 0.003306254 | 114.0551624 | C_5_H_9_NO_2_ | HMDB0000162 | C00148 | ↓ |
| 67 | L-Valine | Amino acids, peptides, and analogues | 2.006009496 | 0.003494484 | 116.0707195 | C_5_H_11_NO_2_ | HMDB0000883 | C00183 | ↓ |
| 68 | L-Lysine | Amino acids, peptides, and analogues | 1.915344663 | 0.002122342 | 145.0973783 | C_6_H_14_N_2_O_2_ | HMDB0000182 | C00047 | ↓ |
| 69 | L-Phenylalanine | Amino acids, peptides, and analogues | 1.901205062 | 0.001419203 | 164.0709717 | C_9_H_11_NO_2_ | HMDB0000159 | C00079 | ↓ |
| 70 | Citrulline | Amino acids, peptides, and analogues | 1.870833747 | 0.002302857 | 174.0877488 | C_6_H_13_N_3_O_3_ | HMDB0000904 | C00327 | ↓ |
| 71 | Pyroglutamic acid | Amino acids, peptides, and analogues | 1.832285076 | 0.000655913 | 128.0344067 | C_5_H_7_NO_3_ | HMDB0000267 | C01879 | ↓ |
| 72 | L-Norleucine | Amino acids, peptides, and analogues | 1.790713057 | 0.002984796 | 130.086402 | C_6_H_13_NO_2_ | HMDB0001645 | C01933 | ↓ |
| 73 | Isobutyrylglycine | Amino acids, peptides, and analogues | 1.767180588 | 0.002683584 | 144.0657236 | C_6_H_11_NO_3_ | HMDB0000730 | Not Available | ↑ |
| 74 | D-Alanine | Amino acids, peptides, and analogues | 1.74166595 | 0.00392005 | 88.03964428 | C_3_H_7_NO_2_ | HMDB0001310 | C00133 | ↓ |
| 75 | LysoPA(16:0/0:0) | Glycerophosphates | 2.03256694 | 9.10723E-05 | 409.2370058 | C_19_H_39_O_7_P | HMDB0007853 | C04036 | ↑ |
| 76 | Cortisone | Hydroxysteroids | 2.015739437 | 0.004890567 | 359.1910373 | C_21_H_30_O_5_ | HMDB0000063 | C00735 | ↓ |
| 77 | Allantoin | Imidazoles | 1.985753259 | 0.000169816 | 157.0359053 | C_4_H_6_N_4_O_3_ | HMDB0000462 | C01551 | ↑ |
| 78 | (Â±)-Tryptophan | Indolyl carboxylic acids and derivatives | 1.771633643 | 0.003938438 | 203.0823175 | C_11_H_12_N_2_O_2_ | HMDB0030396 | C00806 | ↓ |
| 79 | Isohyodeoxycholic acid | Bile acids, alcohols and derivatives | 1.746538319 | 0.002050486 | 391.2868312 | C_24_H_40_O_4_ | HMDB0000664 | Not Available | ↑ |
| 80 | 1,3,5-Trihydroxybenzene | Benzenetriols and derivatives | 1.676736261 | 0.008235941 | 125.0233741 | C_6_H_6_O_3_ | HMDB0013675 | C02183 | ↑ |
| 81 | 15-Keto-prostaglandin E2 | Eicosanoids | 1.517765333 | 0.001114381 | 349.2056204 | C_20_H_30_O_5_ | HMDB0003175 | C04707 | ↑ |
