# Supplementary material for: Ameliorating potential of Auricularia auricula-judae polysaccharides in mitigating hypercaloric diet-induced behavioral disorders through gut microbiota regulation
Source: Front Nutr. 2025 May 29;12:1585778. doi: 10.3389/fnut.2025.1585778 (PMC12158723; doi:10.3389/fnut.2025.1585778)
Supplement: Supplementary file 2 [file Image_1.pdf]

Supplementary Material

Supplementary Figure 1

Monosaccharide composition of (A) Standard samples and (B) AAP were determined using high-performance liquid chromatography (HPLC). Weigh 4 mg of polysaccharide in 5 mL of acid water tube, and add 1.7 mL of double-distilled water to dissolve. After the polysaccharide was completely dissolved, 0.3 mL of trifluoroacetic acid was added and placed in an oven at 121°C for 2 h. After removing the trifluoroacetic acid to neutrality, 2 mL of double-distilled water was added, and the solution was spin evaporated at 45°C until it was odorless. A 0.5 mol/L PMP methanol solution and a 0.3 mol/L NaOH solution were prepared to provide an alkaline environment. Add 200 μL of 0.3 mol/L NaOH solution and 200 μL of 0.5 mol/L PMP methanol solution to dissolve fully by shaking, and then react in a water bath at 70°C for 1.5 h. After the reaction, add glacial acetic acid to neutralize the reaction to a weak acidity, and then add an equal volume of dichloromethane to extract the centrifuge and add to the supernatant, and add to the glacial acetic acid to adjust the pH to 4. Add dichloromethane to extract 2-3 times, concentrate under reduced pressure at 39°C, add 1 mL of water to redissolve, and pass through the activated C18 extraction column. Add 10 mL of distilled water to remove PMP and salt, then elute with 25% acetonitrile and take over 3 mL. The eluate was passed through 0.22 μm organic filtration membrane and put into liquid-phase vials, and stored at -20°C for spare parts.

The liquid-phase conditions for the monosaccharides were as follows: SinoChron ODS-BPC18 column (4.6×250 mm), flow rate of 0.3 mL/min, elution time of 40 min, column temperature of 30°C, injection volume of 10 μL, detection wavelength of 254 nm, and mobile phase: acetonitrile-ammonium acetate solution (18.5:81.5, v/v).

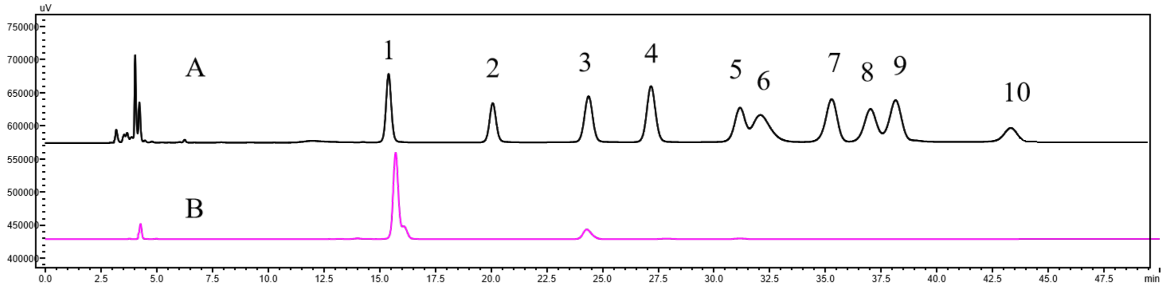

Supplementary Figure 1 Monosaccharide chromatogram of AAP
(A) Standard sample; (B) AAP.
1-10 in the standard samples represent mannose (Man), rhamnose (Rha), glucuronic acid (GlcA), galacturonic acid (GalA), glucoacetylgalactose (GalNAc), galactose (Gal), xylose (Xyl), arabinose (Ara) and fucose (Fuc), respectively.

Supplementary Figure 2

The molecular weight of AAP was analyzed by HPLC. 0.02 M phosphate buffer solution, pH 6.0, was prepared, filtered and ultrasonicated for 20 min. 0.1 mol/L phosphate buffer solution was prepared, and the pH was adjusted to 6 by the addition of 0.1% Na_2SO_4 and 0.05% NaN_3, and then filtered and ultrasonicated for 20 min. 2 mg of the sample was weighed, and then 1 mL of buffer salts was added. The supernatant was centrifuged 4 times, passed through a 0.22 µm filter membrane and put into a liquid-phase vial. The dextran standard was injected according to the molecular weight, the horizontal coordinate of the standard curve was the logarithm of molecular weight, lg MW, and the vertical coordinate was the retention time, and the linear regression equation of molecular weight was plotted. Pipette 0.5 mL of 0.2 mg/mL AAP solution was operated according to the above steps, and the average molecular weight was calculated according to the standard curve equation. The equation of the molecular weight standard curve was y=-0.1724x+9.7135, R^2=0.9946. The peak times of AAP samples were substituted into the equation respectively, and the average molecular weight of AAP was calculated to be 1.91×10^3 kDa.

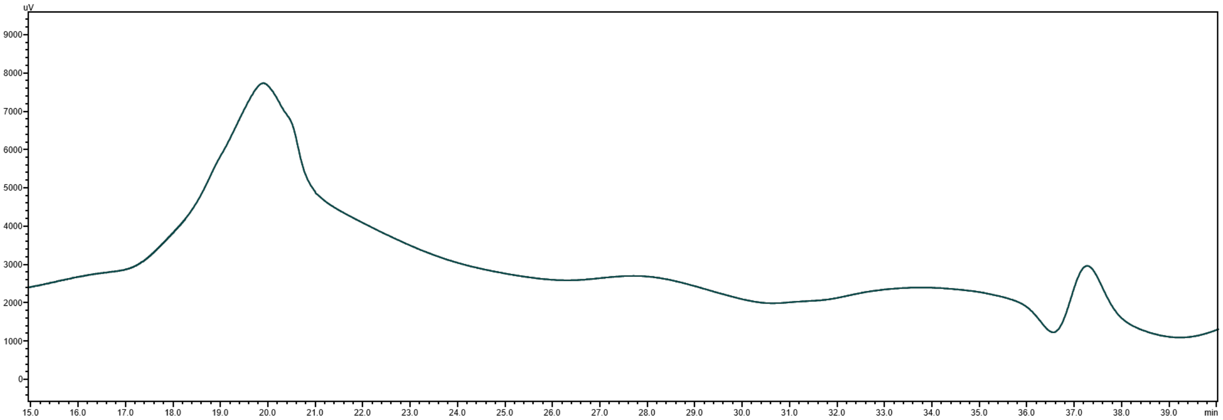

Supplementary Figure 2 Molecular weight distribution of AAP

Supplementary Figure 3

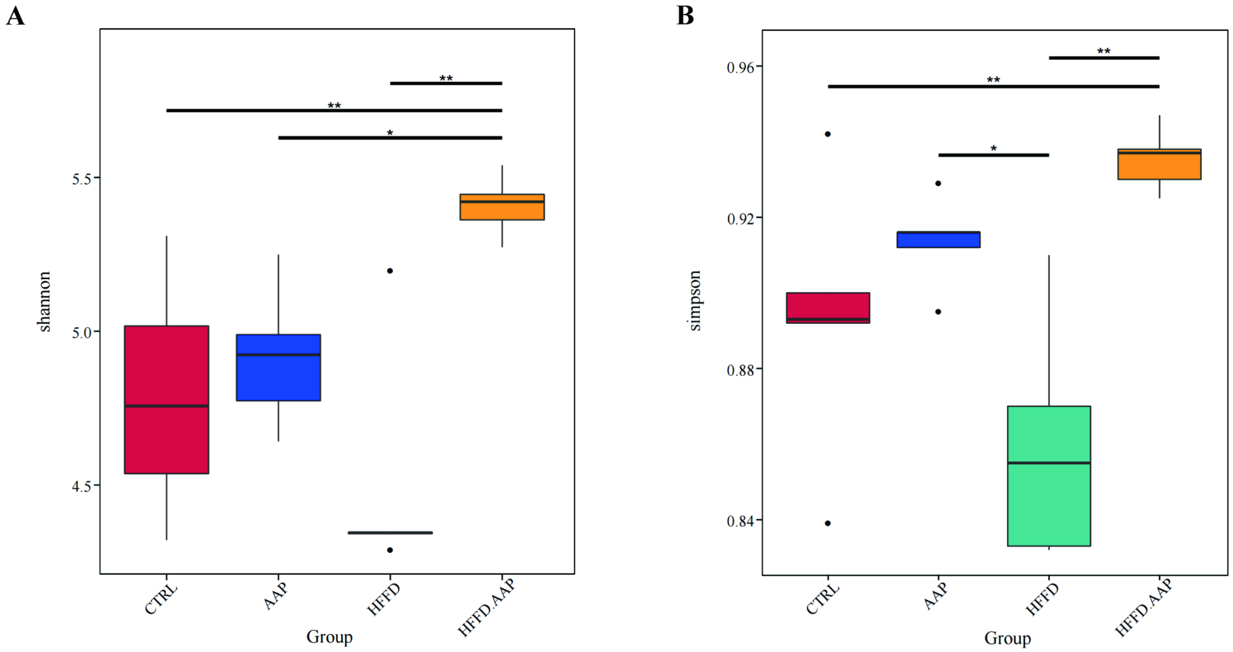

Supplementary Figure 3 The effects of Auricularia auricula polysaccharides on α diversity of intestinal flora in mice.
(A) Shannon index; (B) Simpson index.
